# Implementation of Primary Immune Thrombocytopenia Clinical Practice Guidelines for Management of Pregnancy

**DOI:** 10.3390/jcm13216477

**Published:** 2024-10-29

**Authors:** Amanda J. Llaneza, Laura A. Beebe, Janis E. Campbell, Marshall K. Cheney, Ying Zhang, Deirdra R. Terrell

**Affiliations:** 1 Department of Biostatistics and Epidemiology, Hudson College of Public Health, University of Oklahoma Health Sciences Center, Oklahoma City, OK 73104, USA; amanda.llaneza@mssm.edu (A.J.L.); laura-beebe@ouhsc.edu (L.A.B.); janis-campbell@ouhsc.edu (J.E.C.); ying-zhang4@ouhsc.edu (Y.Z.); 2 Department of Health and Exercise Science, University of Oklahoma, Norman, OK 73019, USA; marshall@ou.edu

**Keywords:** primary immune thrombocytopenia, ITP, clinical practice guidelines, pregnancy

## Abstract

**Background:** Managing primary immune thrombocytopenia (ITP) in pregnancy is challenging. Providers must balance bleeding risk against medication toxicity. The evaluation of the implementation of pregnancy-specific ITP clinical guidelines has not been widely studied. The goal of this study was to describe the implementation of pregnancy-specific ITP guidelines at an academic health center. **Methods:** We conducted a retrospective chart review at the University of Oklahoma Health system from 2011 to 2020. Descriptive statistics were calculated to summarize the characteristics of the study population. Management, according to the clinical guidelines (American Society of Hematology; American College of Obstetricians and Gynecologists) was evaluated during pregnancy and during/for delivery. **Results:** A total of 85 pregnant persons with ITP were included. The majority (68%; 58/85) delivered vaginally. There were 0 maternal deaths and 2 infant deaths. No patients had major bleeding during pregnancy. Postpartum hemorrhage was experienced by 14%. The management of thrombocytopenia during pregnancy was 100% adherent to the strong recommendation for severe (n = 13) and mild (n = 11) thrombocytopenia. However, 18/50 (36%) asymptomatic persons with moderate thrombocytopenia received treatment despite the strong recommendation that treatment was unnecessary. Additionally, 8/21 (38%) persons with moderate thrombocytopenia received treatment to increase platelet counts for epidural anesthesia despite the guideline’s suggestion that it was unnecessary. **Conclusions:** During pregnancy, patients with severe thrombocytopenia (i.e., most at risk of bleeding) received treatment. On the other hand, approximately 40% of pregnant persons with ITP received unnecessary treatment for moderate asymptomatic thrombocytopenia either during pregnancy or for an epidural. Utilizing clinical practice guidelines would reduce the overtreatment of pregnant persons which would reduce the potential side effects of therapy for the mother and infant.

## 1. Introduction

Primary immune thrombocytopenia (ITP) is an autoimmune disorder characterized by isolated thrombocytopenia caused by platelet destruction and impaired platelet production which may increase a person’s risk of bleeding [1,2,3]. Disease severity can be defined as mild, moderate, or severe and is associated with the degree of thrombocytopenia, which is a surrogate for the risk of bleeding [4]. ITP is a diagnosis of exclusion, meaning that other causes of thrombocytopenia must be ruled out before a diagnosis can be made [4]. In adults, ITP has an incidence of two to four per 100,000 adults per year [5,6] and a prevalence of 12.1 per 100,000 adults [7]. ITP has a greater prevalence in women than men during the reproductive years of age at 30–40 years [7]. Although ITP is managed by hematologists, pregnancies with ITP must be carefully monitored and should include management with a multidisciplinary team involving the obstetrician/gynecologist, hematologist, and possibly a neonatologist [8].

Hematologists are often consulted regarding appropriate treatment during pregnancy and for labor and delivery for persons with ITP. Patients are often jointly managed with an obstetrician/gynecologist throughout pregnancy and delivery. Blood loss at delivery has been reported to be significantly higher in those who receive treatment for ITP compared to those who do not receive treatment [9]. Heavy bleeding and complications during delivery can be life-threatening for both the pregnant person and the fetus. Essential characteristics to monitor include the severity of thrombocytopenia, bleeding symptoms, and gestational age. During pregnancy, the goal of treatment is to minimize the risk of bleeding while also reducing the risk of adverse effects to treatment for the pregnant person and fetus [10]. For example, although glucocorticoids are considered safe in pregnancy it is recommended that glucocorticoids be tapered to the lowest possible dose to reduce the risk of maternal comorbidities such as diabetes, hypertension, and excessive weight gain [11,12]. Similarly, intravenous immunoglobulin (IVIg) is generally considered safe in pregnancy, but an increased risk of thrombosis in pregnancy has been reported [11]. Furthermore, rituximab has been reported to increase the risk of neonatal lymphopenia [11,13]. Data on the use of thrombopoietin receptor agonists (TPO-RAs) during pregnancy are still emerging; however, studies show a positive response in ITP in pregnancy. The concern with the use of TPO-RAs in pregnancy is that medications such as romiplostim and eltrombopag likely cross the placenta [11,14,15,16]. Additionally, anti-D has been reported to be effective in improving thrombocytopenia during pregnancy, but there is the risk of both maternal and fetal hemolysis, which is contingent on RhD status [14]. This highlights the critical importance of avoiding unnecessary exposure to treatment for the pregnant person or fetus. In fact, it is recommended to avoid unnecessary treatment of asymptomatic patients with milder degrees of thrombocytopenia [8].

Clinical practice guidelines are evidence-based recommendations for diagnosing and treating a medical condition [17]. The integrity of guideline recommendations is based on current medical and research knowledge, and the goal is for the recommendations to be applied in daily medical practice [17]. The goal is for guideline recommendations to be actionable statements that can be used by health professionals to guide treatment decisions [18,19]. Guidelines are created by a panel of experts who summarize the current medical and research knowledge on a condition, explain the benefits and harms of diagnostic procedures and treatments, and provide specific recommendations based on that information [18]. Clinical practice guidelines provide scoring systems or “levels” of confidence on each recommendation provided. Although clinical practice guideline recommendations are not legally binding for health personnel to follow, deviations from strong recommendations should be justified [17]. Best practices also state there should be transparent management of guideline panel members’ conflicts of interest [18,20]. There are two clinical practice guidelines addressing the management of ITP during pregnancy and delivery: (1) the American Society of Hematology (ASH) clinical practice guidelines published in 2011 [8] (the 2019 ASH ITP guidelines do not address pregnancy) and (2) the American College of Obstetricians and Gynecologists (ACOG) guidelines published in 2019 [10].

To date, there is no published literature describing the implementation of guidelines for pregnant patients with ITP. Describing the implementation of clinical guideline recommendations is an important part of evidence-based medicine and identifying potential challenges and opportunities. The goal of this study was to describe the implementation of pregnancy-specific ITP guidelines and management of pregnancies at an academic health center.

## 2. Materials and Methods

### 2.1. Study Design

This study is a retrospective chart review of persons with ITP who delivered a baby within the University of Oklahoma (OU) Health system between 1 November 2011 and 31 December 2020. This included those who had multiple births at OU Health within this timeframe.

### 2.2. Inclusion Criteria

Patients were initially identified as having a billing code for ITP and had also delivered a baby at OU Health during the study timeframe. The eligibility criteria included the following: (1) International Classification of Diseases (ICD)-9 code 287.30 or 287.31 (1/2011–9/2015) or ICD-10 code D69.3 (10/2015–12/2020), (2) delivered a baby at OU Health between 1 November 2011, and 31 December 2020, and (3) aged 15 to 50 years old at the time of delivery.

### 2.3. Primary ITP

Primary ITP is a diagnosis of exclusion which means other known causes of thrombocytopenia should be ruled out. In this study, to ensure pregnant patients with secondary thrombocytopenia (thrombocytopenia that occurred as a result of their primary diagnosis) were not included as ITP, a manual chart review was also conducted. Additionally, although being billed for an ITP diagnosis was an inclusion criterion, previous studies have reported the positive predictive value of the ICD billing code for ITP to only be between 65 and 71% [21,22]. Therefore, the manual review of the medical record was the final determination to either confirm or rule out an ITP diagnosis.

Patients were determined to have primary ITP if a hematologist consultation note in the medical chart confirmed the diagnosis of ITP either during the current pregnancy or plus or minus one year of the delivery date. For persons without evidence of a hematology consultation in their chart, a diagnosis of primary ITP was determined if they had a recorded platelet count <100 × 10^9^/L at any time during the current pregnancy or at the time of delivery without a known cause for the thrombocytopenia documented in the medical record. This platelet count threshold was determined based on a previous study by Reese et al., which reported that a platelet count <100 × 10^9^/L was more aligned with a diagnosis of ITP versus gestational thrombocytopenia [23].

Upon medical chart review, if a patient was determined to have secondary thrombocytopenia (i.e., evidence of an alternative diagnosis known to be associated with thrombocytopenia written in the medical record) they were then excluded. However, if a patient had evidence of both ITP and an additional diagnosis, then the medical record was reviewed to determine if the ITP preceded the additional diagnosis, and if so, the patient was classified as primary ITP and included.

### 2.4. Data Collection

Data were abstracted from patients’ medical records using a structured form. Variables included maternal age, maternal race/ethnicity, gestation time in weeks at delivery, platelet count at delivery, bleeding events during pregnancy (classified as either major or minor according to the International Society on Thrombosis and Hemostasis definitions), [24] treatment administered for thrombocytopenia, indication for treatment (i.e., vaginal bleeding), mode of delivery, type of anesthesia, postpartum hemorrhage (defined as >500 mL after vaginal delivery and >1000 mL after cesarean delivery), infant birthweight, and infant status (dead or alive).

The lowest platelet count during the pregnancy was recorded, and the thrombocytopenia was classified as either mild (platelet count ≥100 × 10^9^/L and <150 × 10^9^/L), moderate (platelet count ≥30 × 10^9^/L and ≤99 × 10^9^/L), or severe (platelet count <30 × 10^9^/L) [8].

### 2.5. Clinical Practice Guideline Definitions

The implementation of ITP-specific pregnancy clinical guidelines were determined for the ASH ITP guidelines published in 2011 and the ACOG guidelines on thrombocytopenia published in 2019 [8,10]. The 2011 ASH guidelines used the Grading of Recommendations, Assessment, Development, and Evaluations (GRADE) methodology and reported recommendations as either a strong recommendation or a conditional suggestion based, in part, on the quality and strength of the evidence [8,17,25]. Strong recommendations should be interpreted by clinicians as the recommended clinical course of action for most patients. With a strong recommendation, the desirable consequences of the treatment outweigh the undesirable consequences, and it is believed that most patients would want to follow the recommended management plan [17,25]. Conditional suggestions should be interpreted by clinicians as those that should be presented to the patient in such a way that it is a potential treatment option after taking into account the patient’s values and preferences (i.e., shared decision-making). A conditional suggestion conveys that there is an unclear or close balance between the desirable and undesirable effects of the medication in a condition or scenario. It is believed that although many patients would want the suggested management plan, some patients would refuse it due to differences in individual values and preferences [17,25].

The ACOG 2019 guidelines used a grading system methodology with three levels (Levels A, B, and C) [10]. Level A was the highest recommendation level and based on good and consistent scientific evidence. Level B recommendations were based on limited or inconsistent scientific evidence, and Level C recommendations were based on consensus and expert opinion [10]. For this study, we determined that ACOG recommendations designated as ‘A’ were consistent with an ASH ‘strong’ recommendation and ACOG levels B and C were consistent with ASH ‘conditional’ suggestions.

### 2.6. Guideline Evaluation

The management of thrombocytopenia according to the clinical guidelines was evaluated for pregnant persons at two different time points: (1) recommended management during the pregnancy, and (2) recommended management during or for delivery. Strong recommendations were classified as either adherent (yes) or not adherent (no) to the recommendation, and conditional suggestions were classified as either consistent with (yes) or not consistent with (no) the suggestion (Table 1).

If the two clinical guidelines disagreed on the level of the recommendation, then preference was given to the highest level. For example, if one guideline had a recommendation as ‘strong’ and the other guideline stated the same recommendation was ‘conditional’, then it was evaluated as a ‘strong’ recommendation.

### 2.7. Ethics Approval

This study was approved by the Institutional Review Board (IRB) of the University of Oklahoma Health Sciences Center (IRB #12910 on 21 January 2021).

### 2.8. Statistical Analysis

Descriptive statistics were calculated to summarize the demographic characteristics of the study population. The mode of delivery was described. For strong recommendations, we described the proportion of patients who were treated in adherence to the strong recommendation versus those who were not treated in adherence to the strong recommendation. If the patient received treatment when the guideline stated it was unnecessary, this was also accounted as nonadherent. For conditional suggestions, we described the proportion of patients who were treated following conditional suggestions versus those who were not treated according to the conditional suggestions.

Management according to the guidelines was described during pregnancy related to bleeding events and the severity of thrombocytopenia, during delivery, and for epidural/spinal anesthesia. Descriptive statistics were calculated using SAS, version 9.4 (SAS Institute, Inc., Cary, NC, USA).

## 3. Results

**Figure 1:**
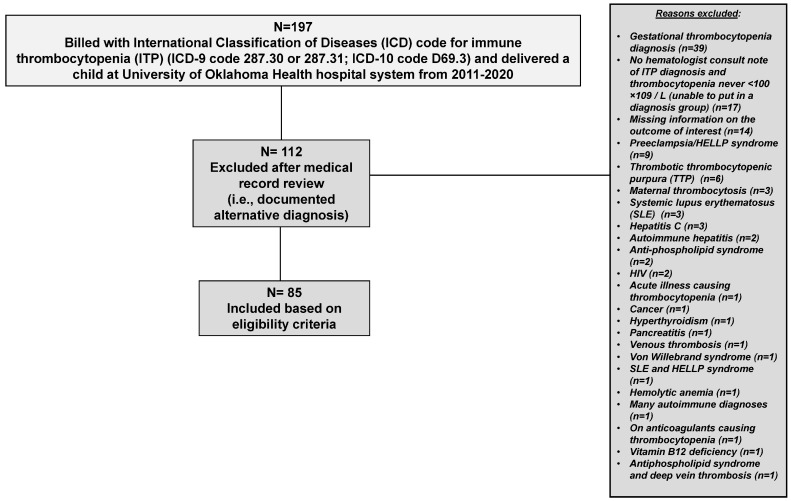
Patient flow chart of pregnant people with primary immune thrombocytopenia (ITP) who delivered at the University of Oklahoma Health hospital system 2011–2020.

A total of 197 patients had an ICD code for ITP and delivered a baby within the OU Health system between 1 November 2011 and 31 December 2020. Of those 197 patients, 112 (57%) were excluded following manual review of the medical record. Thirty-one patients were excluded due to insufficient documentation in the medical record to confirm or rule out ITP (Figure 1). The remaining 81 patients were excluded from further analysis due to a documented explanation or condition on the medical chart as a cause for their thrombocytopenia (i.e., the patient had secondary thrombocytopenia). Of the eighty-one excluded patients, thirty-nine patients had a documented diagnosis of gestational thrombocytopenia as a reason for their thrombocytopenia, nine patients had preeclampsia/HELLP syndrome documented, six patients had immune-mediated thrombotic thrombocytopenic purpura (TTP), three patients had maternal thrombocytosis, three patients had systemic lupus erythematosus (SLE), three patients had hepatitis C, two patients had autoimmune hepatitis, two patients had anti-phospholipid syndrome, two had human immunodeficiency virus (HIV), and the remaining twelve patients had various conditions, as shown in Figure 1. Therefore, 85 pregnant patients with ITP were included in this study (Figure 1).

The mean age of pregnant persons with ITP was 28 years (range 17 to 39 years). The majority 48/85 (57%) were White, 6/85 (7%) were Black, 2/85 (2%) were American Indian/Alaska Native, 4/85 (5%) were Asian, and 25/85 (29%) were Hispanic. For those who identified as Hispanic, the medical records did not indicate another race or ethnicity. The mean gestation at birth was 37.8 weeks, and the majority 58/85 (68%) of persons delivered vaginally (Table 2). For the majority of mothers (60/85; 71%), this was their first pregnancy.

There were two infant deaths recorded at delivery. One infant was a triplet who was delivered prematurely. The second infant death was an intrapartum fetal death resulting from severe preeclampsia in the mother. This mother had a diagnosis of ITP for several years prior to becoming pregnant. There were zero maternal deaths. Additionally, there was no evidence of a major bleeding event occurring during pregnancy (0/85). However, 6/85 (7%) experienced one minor bleeding event, and 2/85 (2%) experienced more than one minor bleeding event during pregnancy. For the majority (53/85, 62%) of persons the severity of the lowest thrombocytopenia that occurred during pregnancy was classified as moderate (platelet count ≥30 × 10^9^/L and ≤99 × 10^9^/L). At delivery, 12/85 (14%; 95% confidence interval (CI) 7%, 23%) experienced postpartum hemorrhage. Postpartum hemorrhage was managed with carboprost tromethamine, misoprostol, and/or methylergonovine, or a combination of those treatments (Table 2). Among those 12, 10/12 (83%) were vaginal deliveries and 2/12 (17%) were cesarean deliveries.

### 3.1. During Pregnancy: Implementation of Guidelines

Both guidelines stated that treatment should be initiated for symptomatic bleeding or severe thrombocytopenia (platelet count < 30 × 10^9^/L) during pregnancy. This recommendation was strong for ASH and conditional for ACOG (Table 1) and, therefore, evaluated as a strong recommendation. ASH also stated that when treatment was initiated it should consist of corticosteroids or intravenous immune globulin (IVIg).

**Figure 2:**
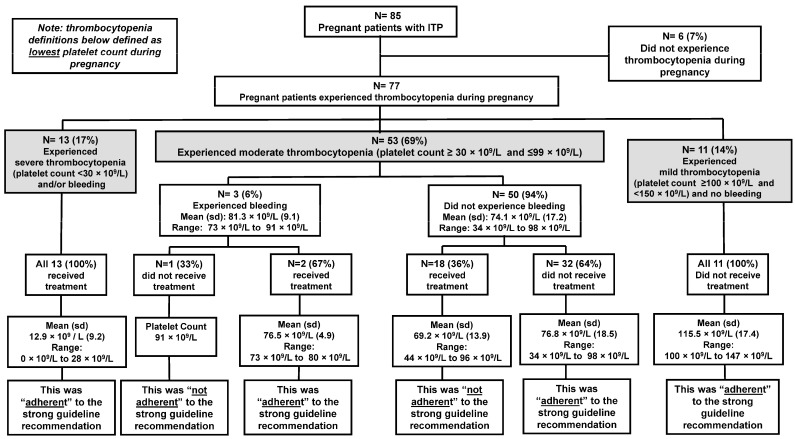
Adherence to strong guideline recommendation for management of thrombocytopenia during pregnancy for persons with primary immune thrombocytopenia (ITP) at the University of Oklahoma Health hospital system 2011–2020.

There were 77/85 (91%) pregnant people who experienced thrombocytopenia during pregnancy (Figure 2). The 6/85 (7%) pregnant people who did not experience thrombocytopenia during their pregnancy all had a history of ITP prior to pregnancy and either had a splenectomy or were on long-term (>10 years) management with corticosteroid treatment. Among the 77 pregnant people who experienced thrombocytopenia, the mean age of those who received treatment was 27.8 years compared to 28.2 years for those who did not receive treatment.

Only 13 persons (17%) experienced severe thrombocytopenia (platelet count <30 × 10^9^/L) during their pregnancy, and the mean platelet count was 12.9 × 10^9^/L (range: 0 × 10^9^/L to 28 × 10^9^/L). All 13 (100%) of the pregnant people with severe thrombocytopenia received treatment (either corticosteroids or IVIg) for their thrombocytopenia. Therefore, this management was “adherent” to the strong guideline recommendation (Figure 2). Only 5/13 (38%) persons with severe thrombocytopenia experienced symptomatic bleeding. Bleeding events consisted of epistaxis, rectal bleeding, and vaginal bleeding.

There were 53/77 (69%) pregnant people who experienced moderate thrombocytopenia (platelet count ≥30 × 10^9^/L and ≤99 × 10^9^/L) during their pregnancy. The mean platelet count was 81 × 10^9^/L (range: 73 × 10^9^ L to 91 × 10^9^/L). Of this group, only three people experienced symptomatic bleeding, which included vaginal bleeding and epistaxis. Two people with symptomatic bleeding (one experienced two bleeding events during pregnancy) received treatment (either corticosteroids or IVIg), which was “adherent” to the strong guideline recommendation. One person with symptomatic bleeding did not receive treatment which was “not adherent” to the strong guideline recommendation (Figure 2). The majority 50/53 (94%) of people who experienced moderate thrombocytopenia did not experience symptomatic bleeding. For this group, the mean platelet count was 74 × 10^9^/L (range: 34 × 10^9^/L to 98 × 10^9^/L). Of the asymptomatic persons, the majority (32/50; 64%) did not receive treatment, which was “adherent” to the strong guideline recommendation. However, 18/50 (36%) persons who were asymptomatic with moderate thrombocytopenia received treatment for thrombocytopenia when the guidelines deemed treatment unnecessary. This management was considered “not adherent” to the strong guideline recommendation (Figure 2). The type of treatment received consisted of corticosteroids (n = 16), IVIg (n = 1), and a platelet transfusion (n = 1). There were several obstetrician-gynecologists that began patients on treatment to improve moderate thrombocytopenia in the third trimester of pregnancy. Although the indication for treatment was not always noted in the medical record, several times it was stated that a higher platelet count would allow the patient more options at the time of delivery.

### 3.2. During and for Delivery: Implementation of Guidelines

There were three conditional suggestions related to the management of thrombocytopenia during and for delivery (Table 1). However, one conditional suggestion was not evaluated as there were limited data in the electronic medical records to determine if the mode of delivery was based on obstetric reasons or for other reasons.

The next conditional suggestion stated that treatment should be initiated if the platelet count was <50 × 10^9^/L to improve the thrombocytopenia prior to a cesarean delivery (Table 1). Of the 85 deliveries, there were 58/85 (68%) with a vaginal delivery and 27/85 (32%) delivered via cesarean (Table 2). The mean platelet count for cesarean deliveries was 113 × 10^9^/L (range: 50 × 10^9^/L to 435 × 10^9^/L). According to the medical record, no treatment was given with the sole intention of raising the platelet count for the cesarean delivery. This management was “consistent with” the conditional suggestion.

**Figure 3:**
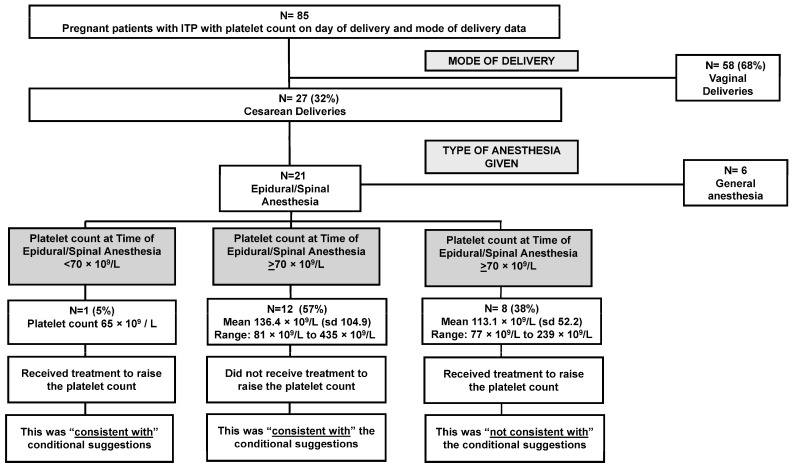
Consistency with conditional recommendations in the management of thrombocytopenia during and for delivery for persons with primary immune thrombocytopenia (ITP) at the University of Oklahoma Health hospital system 2011–2020.

The final conditional suggestion stated that treatment should be initiated for an epidural if the platelet count was <70 × 10^9^/L to improve the thrombocytopenia before the epidural. There were 21 persons who had a cesarean delivery who also received an epidural/spinal anesthesia. There was one pregnant person with a platelet count of 65 × 10^9^/L who received a platelet transfusion to raise their platelet count in order to receive the epidural/spinal anesthesia. Following the platelet transfusion, the platelet count reached 92 × 10^9^/L. This management was “consistent with” the conditional suggestion (Figure 3).

There were 12/21 (57%) people who had a cesarean delivery and epidural/spinal anesthesia with platelet counts at the time of anesthesia greater than the suggested threshold (mean platelet count: 136.4 × 10^9^/L; range: 81 × 10^9^/L to 435 × 10^9^/L). None of the 12 persons received treatment to raise their platelet count before epidural/spinal anesthesia. This management was “consistent with” the conditional suggestion (Figure 3).

However, there were 8/21 (38%) pregnant people who had cesarean deliveries and epidural/spinal anesthesia who received treatment (either IVIg or corticosteroids) to raise their platelet count even though their platelet counts were greater than the suggested threshold of 70 × 10^9^/L (mean platelet count: 113.1 × 10^9^/L; range: 77 × 10^9^/L to 239 × 10^9^/L). This management was “not consistent” with the conditional suggestion (Figure 3). The medical record did not always indicate the reason for treatment in these cases; however, in several medical records, it was noted the patients expressed wanting to receive an epidural and that anesthesiology would require a platelet count ≥100 × 10^9^/L for epidural/spinal anesthesia.

A summary of the guideline results is displayed in Table 3. Adherence to the strong guideline recommendation was 100% for persons with either severe or mild thrombocytopenia. Likewise, 100% of persons who delivered a child via a cesarean were managed consistent with conditional suggestions. However, 36% of pregnant persons with ITP who were asymptomatic (no bleeding symptoms) with moderate thrombocytopenia received medication to raise their platelet count during pregnancy which was not adherent to guideline recommendations. Additionally, 38% of persons who had a cesarean delivery and epidural/spinal anesthesia received treatment to raise their platelet count even though their platelet count at the time of the epidural/spinal anesthesia ranged from 77 × 10^9^/L to 239 × 10^9^/L. This management was not consistent with conditional guideline suggestions.

## 4. Discussion

The implementation of pregnancy-specific guidelines for ITP from ASH and ACOG were evaluated among 85 pregnant people who delivered at an academic health center. Among the 77 patients who experienced thrombocytopenia during pregnancy, the majority (53/77; 69%) experienced moderate thrombocytopenia, while only 13/77 (17%) experienced severe thrombocytopenia.

There was no evidence of a major bleeding event occurring during pregnancy and no maternal deaths. However, delivery with ITP is not without maternal risk. The risk of postpartum hemorrhage in pregnancy in the United States in 2019 was reported to be 4.3% [26]. In comparison, our study found 14% (95% CI 7%, 23%) of the pregnancies were complicated by postpartum hemorrhage. A previous retrospective chart review reported that 33% of women with ITP had postpartum hemorrhage [9]. Furthermore, a study focused on pregnancies with ITP with severe thrombocytopenia (platelet count <50 × 10^9^/L) reported that the incidence of postpartum hemorrhage was 52% [27]. These results suggest that the risk of postpartum hemorrhage in pregnancy in persons with ITP is greater than in the general population.

The management of ITP varies from patient-to-patient due to factors such as the severity of thrombocytopenia, disease duration, quality of life considerations, and access to healthcare for patients [28]. Additionally, management differences may exist between hematologists and obstetricians. Patients in this study who experienced severe thrombocytopenia were managed according to guideline recommendations. However, approximately 40% of pregnant persons with ITP received unnecessary treatment for non-life-threatening thrombocytopenia. Unfortunately, the indication for treatment was not always documented; however, there was evidence to suggest that some obstetricians began prophylactic treatment in the third trimester in preparation for delivery. A previous study conducted in the United Kingdom also reported overtreatment of asymptomatic persons with ITP during pregnancy [27]. Overtreatment is serious as prophylactic treatment with corticosteroids and IVIg comes with potential side effects for the mother and unborn child [27].

Also, 38% of patients who had a cesarean section and epidural/spinal anesthesia received unnecessary treatment to raise their platelet counts at the time of the epidural/spinal anesthesia even with safe platelet count levels. This may indicate that in clinical practice, regardless of guidelines, anesthesiologists may not be comfortable with moderate platelet count thresholds and may require higher platelet count levels for procedures such as epidural/spinal anesthesia.

Strengths to this study included utilizing medical records from a large hospital system in the state of Oklahoma, which captured the diverse treatment experiences of pregnant people with ITP. This study included several years of data (2011–2020) due to the rarity of an ITP diagnosis and added a medical chart review to improve the accuracy of diagnosis. Also, each pregnant individual had an equal time frame (+/−1 year from delivery) for the evaluation of an ITP diagnosis, and this systematic approach minimized bias.

However, data were only captured from encounters within the OU Health hospital system, and it is possible that pregnant people delivered their baby at OU Health but were managed under the care of a community hematologist (i.e., practiced outside of OU Health). As a result, there could be missing information related to the treatment of thrombocytopenia. Furthermore, the goal of conditional guideline suggestions was to incorporate shared decision-making, yet it was not possible to determine from the electronic medical records if the patient’s anxiety was driving the decision to treat non-life-threatening thrombocytopenia versus the physician.

## 5. Conclusions

Overall, those with severe thrombocytopenia and most at risk for bleeding during pregnancy universally received treatment for their thrombocytopenia. On the other hand, approximately 40% of pregnant persons with ITP received unnecessary treatment for moderate asymptomatic thrombocytopenia either during pregnancy or for an epidural. Research has suggested a barrier to implementing clinical guidelines is healthcare practitioners not wanting to alter their practices or not having the infrastructure to implement guidelines [29]. Specifically, the ACOG guidelines state that a platelet count ≥70 × 10^9^/L is safe for anesthesia, but in clinical practice that threshold was higher (≥100 × 10^9^/L). Improved clinical guideline dissemination pathways should be established to ensure that all healthcare practitioners involved in the management of ITP and pregnancy are included. Utilizing current clinical practice guidelines could reduce the overtreatment of pregnant persons and effectively lessen the potential side effects of therapy for the mother and infant.

## Figures and Tables

**Table 1:** Clinical practice guidelines for management of pregnancy in persons with primary immune thrombocytopenia.

Recommended Management	Strength of Recommendation	Society
During Pregnancy		
Platelet count <30 × 10^9^/L or symptomatic bleeding causes the pregnant patient to receive either corticosteroids or intravenous immune globulin (IVIg)	Strong recommendation	American Society of Hematology [8]
Treatment should be initiated if platelet count <30 × 10^9^/L, or if there is symptomatic bleeding, or to increase platelet counts to a level considered safe for procedures	Conditional suggestion	American College of Obstetricians and Gynecologists [10]
During or for Delivery		
Mode of delivery (vaginal vs. cesarean section) should be based on obstetric concerns (platelet count threshold not indicated)	Conditional suggestion	American Society of Hematology [8]
Treatment should be initiated for platelet counts <50 × 10^9^/L for a cesarean delivery	Conditional suggestion	American College of Obstetricians and Gynecologists [10]
Treatment should be initiated for platelet counts <70 × 10^9^/L for an epidural placement	Conditional suggestion	American College of Obstetricians and Gynecologists [10]

**Table 2:** Characteristics of pregnant patients with primary immune thrombocytopenia who delivered at the University of Oklahoma Health hospital system between 2011 and 2020, N = 85.

Characteristics	
	N = 85
Maternal age in years, Mean (std)	28 (4.9)
Gestation time in weeks, Mean (std)	37.8 (3.0)
	n (%)
Maternal Race/Ethnicity	
Non-Hispanic White	48 (57%)
Non-Hispanic Black	6 (7%)
Non-Hispanic American Indian/Alaska Native	2 (2%)
Asian	4 (5%)
Hispanic	25 (29%)
Major Bleeding Event During Pregnancy	
No	85 (100%)
Minor Bleeding Event During Pregnancy	
No	77 (91%)
Yes (1 event)	6 (7%)
Yes (more than 1 event)	2 (2%)
Mode of Delivery	
Vaginal	58 (68%)
Cesarean	27 (32%)
Mode of Delivery	
Neuraxial Anesthesia Given	n = 84
No	23 (27%)
Yes	61 (73%)
Missing	1
Type of Neuraxial Anesthesia Given	n = 61
Spinal	18 (30%)
Epidural	33 (54%)
General Anesthesia	10 (16%)
Postpartum Hemorrhage During Delivery	
No	73 (86%)
Yes	12 (14%)
Type of treatment for hemorrhage	n = 12
Carboprost tromethamine	1 (8%)
Misoprostol	4 (33%)
Carboprost tromethamine and misoprostol	5 (42%)
Methylergonovine, carboprost tromethamine and misoprostol	2 (17%)
Infant Sex Assigned at Birth	n = 86
Male	49 (57%)
Female	37 (43%)
Missing	1
Infant Deaths at Delivery	N = 87
No	85 (98%)
Yes	2 (2%)

**Table 3:** Implementation of clinical practice guidelines for pregnant patients with primary immune thrombocytopenia who delivered at the University of Oklahoma Health hospital system between 2011 and 2020.

	Implementation of Guidelines	
Characteristic	No N (%)	Yes N (%)
During Pregnancy	“Adherent” to strong recommendation	
Severe thrombocytopenia (platelet count <30 × 10^9^/L)	0	13/13 (100%)
Moderate thrombocytopenia (platelet count ≥30 × 10^9^/L and ≤99 × 10^9^/L)	18/53 (36%)	34/53 (64%)
Mild thrombocytopenia (platelet count ≥100 × 10^9^/L and <150 × 10^9^/L)	0	11/11 (100%)
During and For Delivery	“Consistent with” the conditional guidelines	
Cesarean delivery	0	27/27 (100%)
Epidural/spinal anesthesia	8/21 (38%)	13/21 (62%)

## Data Availability

Clinical data cannot be made public due to privacy issues, but limited data can be made available upon special request to the corresponding author with justification for the data request.
